# Impaired Right Ventricular Calcium Cycling Is an Early Risk Factor in R14del-Phospholamban Arrhythmias

**DOI:** 10.3390/jpm11060502

**Published:** 2021-06-03

**Authors:** Kobra Haghighi, George Gardner, Elizabeth Vafiadaki, Mohit Kumar, Lisa C. Green, Jianyong Ma, Jeffrey S. Crocker, Sheryl Koch, Demetrios A. Arvanitis, Phillip Bidwell, Jack Rubinstein, Rutger van de Leur, Pieter A. Doevendans, Fadi G. Akar, Michael Tranter, Hong-Sheng Wang, Sakthivel Sadayappan, Deeptankar DeMazumder, Despina Sanoudou, Roger J. Hajjar, Francesca Stillitano, Evangelia G. Kranias

**Affiliations:** 1 Department of Pharmacology and Systems Physiology, University of Cincinnati College of Medicine, Cincinnati, OH 45267, USA; haghigk@ucmail.uc.edu (K.H.); gardnerg137@gmail.com (G.G.); kumarm3@ucmail.uc.edu (M.K.); green2ls@mail.uc.edu (L.C.G.); majy@ucmail.uc.edu (J.M.); crockejy@mail.uc.edu (J.S.C.); phil@ddt.umn.edu (P.B.); wanghs@ucmail.uc.edu (H.-S.W.); 2 Molecular Biology Division, Biomedical Research Foundation of the Academy of Athens, 11527 Athens, Greece; lvafiadaki@bioacademy.gr (E.V.); arvanitd@bioacademy.gr (D.A.A.); dsanoudou@med.uoa.gr (D.S.); 3 Department of Medicine, University of Cincinnati College of Medicine, Cincinnati, OH 45267, USA; kochse@ucmail.uc.edu (S.K.); rubinsjk@ucmail.uc.edu (J.R.); trantemc@UCMAIL.UC.EDU (M.T.); SADAYASL@ucmail.uc.edu (S.S.); demazudr@ucmail.uc.edu (D.D.); 4 Netherlands Heart Institute, Utrecht and Department of Cardiology, University Medical Center Utrecht, 3581 HG Utrecht, The Netherlands; R.R.vandeLeur-3@umcutrecht.nl (R.v.d.L.); P.Doevendans@umcutrecht.nl (P.A.D.); 5 Cardiovascular Research Center, Icahn School of Medicine at Mount Sinai, New York, NY 10029, USA; fadi.akar@yale.edu (F.G.A.); francesca.stillitano@mssm.edu (F.S.); 6 Center for New Biotechnologies and Precision Medicine, Medical School, National and Kapodistrian University of Athens, 11517 Athens, Greece; 7 Phospholamban Heart Foundation, Postbus 66, 1775 ZH Middenmeer, The Netherlands; rhajjar@me.com

**Keywords:** arrhythmia, calcium, phospholamban, mutant

## Abstract

The inherited mutation (R14del) in the calcium regulatory protein phospholamban (PLN) is linked to malignant ventricular arrhythmia with poor prognosis starting at adolescence. However, the underlying early mechanisms that may serve as prognostic factors remain elusive. This study generated humanized mice in which the endogenous gene was replaced with either human wild type or R14del-PLN and addressed the early molecular and cellular pathogenic mechanisms. R14del-PLN mice exhibited stress-induced impairment of atrioventricular conduction, and prolongation of both ventricular activation and repolarization times in association with ventricular tachyarrhythmia, originating from the right ventricle (RV). Most of these distinct electrocardiographic features were remarkably similar to those in R14del-PLN patients. Studies in isolated cardiomyocytes revealed RV-specific calcium defects, including prolonged action potential duration, depressed calcium kinetics and contractile parameters, and elevated diastolic Ca-levels. Ca-sparks were also higher although SR Ca-load was reduced. Accordingly, stress conditions induced after contractions, and inclusion of the CaMKII inhibitor KN93 reversed this proarrhythmic parameter. Compensatory responses included altered expression of key genes associated with Ca-cycling. These data suggest that R14del-PLN cardiomyopathy originates with RV-specific impairment of Ca-cycling and point to the urgent need to improve risk stratification in asymptomatic carriers to prevent fatal arrhythmias and delay cardiomyopathy onset.

## 1. Introduction

Arrhythmogenic cardiomyopathy (ACM) is a rare disease with a prevalence of about 1:1000 to 1:5000. Early characteristics include predisposition to ventricular arrhythmias even in the absence of overt functional or structural abnormalities. At later stages, clinical manifestations are associated with the development of progressive heart failure [1,2]. ACM is mainly a genetically linked disease and a key etiology of sudden cardiac death in young carriers, usually linked to exercise or adrenergic stimulation [2]. The underlying cellular etiologies are not well defined and delineation of the early pre-pathogenic mechanisms for preventative care remains a challenge. Although ACM is mainly associated with mutations in desmosomal genes, it is becoming increasingly apparent that non-desmosomal proteins may also lead to similar clinical phenotypes [1]. Indeed, mutations in genes that regulate calcium homeostasis, such as ryanodine receptor and phospholamban (PLN), have been shown to result in ACM [3,4,5]. A mutation in PLN, entailing deletion of Arg-14 (R14del), a highly conserved basic amino acid in the coding region, has been receiving interest recently as carriers develop abnormal characteristic electrocardiogram (ECG) with an increased risk for malignant ventricular arrhythmias at an early age [3,5]. PLN is a prominent regulator of SR Ca-cycling and contractility. In the dephosphorylated state, PLN is an inhibitor of the Ca-affinity of SERCA2a, and upon its phosphorylation by β-adrenergic agonists, the inhibitory effects are relieved [6]. The R14del-PLN mutation has only been found in the heterozygous state, and carriers have been identified in several European countries, US and Canada [3,7]. The carriers have prompted the formation of the PLN Foundation [8] as prognosis remains poor, starting at late adolescence, and mortality is usually observed between the ages of 25–35 years [3,5,7]. Cardiomyocytes, derived from human R14del-PLN induced pluripotent stem cells (iPSC-CMs), displayed irregular Ca-handling [9], but the contribution of this mutation to ACM remains elusive.

Previous overexpression studies of R14del-PLN in either heterologous systems or in mouse models indicated super-inhibition of SERCA2a activity that led to cardiac remodeling and early death [3]. Interestingly, when the R14del-PLN was expressed in the PLN-deficient background, the mutant did not localize to SR and there was no inhibition of SERCA2a activity [10]. Recently, a mouse model with insertion of the R14del mutation in the endogenous PLN locus was generated, and while homozygous mice died young with dilated cardiomyopathy (DCM), heterozygous mice showed a propensity to arrhythmia only at an old age (18 months) [11]. However, these findings do not reflect the human phenotype, since there are no homozygous R14del-PLN patients identified and the heterozygous carriers exhibit abnormal ECG characteristics even at a young age [3,5]. In addition, human PLN contains Lys instead of Asn at position 27, and this introduces an additional positive charge in the inhibitory domain of PLN, consisting of amino acids (AA) 21–30, which may alter the structure of the protein. Indeed, previous NMR studies of AA 1–36 suggested that the spatial conformation of human PLN is significantly different from that in mouse or other mammalian species [12]. Furthermore, Asn27 in PLN was reported to interact with Leu321 in SERCA2a, and alterations in this residue are expected to have significant effects on the SERCA2a affinity. Indeed, cardiac overexpression of human PLN in the mouse heart resulted in increased inhibitory activity compared to mouse PLN [12].

Thus, there is a pressing need to better understand the molecular mechanisms associated with the human R14del-PLN mutation and develop a mechanistic insight of potential Ca-cycling defects that precede structural alterations, which may have important therapeutic implications. In this regard, we generated humanized mouse models in which the endogenous PLN was replaced by human wild type (WT-PLN) or human R14del-PLN in the heterozygous state, to mimic the human carriers. Our findings indicate that R14del-PLN was associated with infrequent premature ventricular contractions under basal conditions, while stress caused delayed ventricular activation, prolonged repolarization, and a high burden of premature ventricular contractions and ventricular tachyarrhythmia, originating from the right ventricle in vivo. At the cellular level, action potential duration was prolonged, Ca-cycling was depressed, and contractile parameters were inhibited only in right ventricular mutant myocytes. These findings reveal that RV-specific Ca-impairment is an early defect associated with R14del-PLN mutation and point to the importance in stratification of asymptomatic carriers to prevent the risk of sudden death and/or delay overt cardiac disease.

## 2. Materials and Methods

### 2.1. Humanized WT-PLN and R14del-PLN Knock-in Mice

Mice harboring the human WT and R14del-PLN coding sequence were created by inserting LoxP-H2B-GFP-4XpolyA-FRT-Neo-FRT-LoxP-hPLN^WT/R14del^ cassette into the PLN start codon at exon 2 through gene targeting. Chimeric males (129/Sv-CP; C57Bl/6J) were bred to Sox2-Cre mice to establish a hybrid line for WT and R14del mice. The animals were genotyped by tail PCR using a primer set (forward: AAGCTGTGCATATCTACCCAGAGA; reverse: AGCTGAGTTGGCATGTTGCAGGT) to amplify a ~500 bp fragment containing the human coding sequence of PLN. To confirm the presence of the mutation, the PCR product was purified and sequenced by Sanger sequencing. To establish the R14del heterozygous colony, the WT homozygous mice were crossed with R14del homozygous mice. The progenies were genotyped using isolated tail DNA samples and PCR methodology. The male heterozygous mice (R14del/WT), as well as WT controls, were used for experimental studies. Reagents’ usage, animal procedures, and animal care were approved by the Institutional Animal Care and Use Committee of the University of Cincinnati. The investigation followed the guidelines by Association for Assessment and Accreditation of Laboratory Animal Care and Guide for the Care and Use of Laboratory Animals by National Institutes of Health. Animals were euthanized by carbon dioxide (CO2) inhalation followed by cervical dislocation.

### 2.2. Histology

Hearts were excised from anesthetized (Euthasol, 200 mg/kg ip, Virbac AH, Inc., Fort Worth, TX, USA) 12-week-old mice (WT, n = 6; R14del, n = 6) and immediately fixed in 10% neutral buffered formalin (Sigma, Saint Loius, MO, USA). Paraffin-embedment cardiac sections were prepared at 5 μm of thickness. Sections were deparaffinized with two washes in fresh xylene for five minutes and then rehydrated through a series of washes in decreasing concentrations of ethanol (100%, 95%, 80%, 70%, 50%) for 5 min at each step. Following rehydration, sections were incubated in 0.1% Sirius red in saturated picric acid for 1 h, then washed in acidified water. The picrosirius red-stained sections of the hearts were checked for the presence of fibrosis. Fast Green solution was used in combination with the picrosirius red, which stains non-collagenous proteins. Whole-heart images were obtained using Axio Scan.Z1 (Zeiss).

### 2.3. In Vivo Stress-Induced Arrhythmias

Mice were anesthetized using 2.5% Avertin (2,2,2 Tribromoethanol, Sigma; 100 mg/kg, ip). Avertin was selected as anesthetic reagent because it is appropriate for acute and short experiments in mice as well as due its ability to maintain stable cardiac rhythmic function (physiological blood pressure, heart rate and no bradycardic effect), compared to ketamine or ketamine/xylazine mix [13,14,15]. After stabilization and a baseline ECG recording for 5 min, a simultaneous intraperitoneal injection of caffeine (20 mg/kg, Sigma) and epinephrine (2 mg/kg, Sigma) was administered, which was followed by a subsequent recording period of 10–20 min. An equivalent of lead I and II surface ECG recording (PowerLab, AD Instruments, Colorado Springs, CO, USA) was performed. Image processing and data analysis were performed using LABCHART software (AD Instruments, Colorado Springs, CO, USA). The ECG waveforms’ morphology was manually reviewed and analyzed using custom software (MathWorks, Inc., Natick, MA, USA). 

### 2.4. Human R14del-PLN Electrocardiogram Studies

From the dataset of all resting 12-lead ECGs acquired in the nursing wards or outpatient clinic of the University Medical Center Utrecht (UMCU) from January 1991 until December 2020, 105 R14del-PLN patients were identified. ECGs were obtained using General Electric MAC 5500 (GE Healthcare, Chicago, IL, USA), and only the first ECG was included. All ECGs of insufficient quality, with supra (ventricular) arrhythmias or paced rhythms or acquired after implantation of a left ventricular assist device (LVAD) or heart transplantation were excluded. Conduction intervals and median beat ECGs were extracted from the MUSE ECG system and used to calculate the peak R-wave voltage in the extremity leads using in-house software. Extracted data were de-identified in accordance with EU General Data Protection Regulation. Thus, written informed consent was not required by the ethical committee.

### 2.5. Electrophysiological Recordings

Isolated RV and LV myocytes from WT-PLN and R14del-PLN hearts were perfused with Tyrode’s solution containing (in mM): NaCl 140, KCl 5.4, MgCl_2_ 1, CaCl_2_ 1.8, Hepes 5, and glucose 10 (PH = 7.4). Action potentials were recorded using whole-cell patch clamp recording under current clamp mode and were triggered with just-threshold 2 ms current steps at a stimulation rate of 1 Hz at room temperature (24 ℃). Glass pipettes were filled with a solution containing (in mM) K-aspartate 110, KCl 20, EGTA 10, Hepes 10, MgCl_2_ 2.5, NaCl 8, CaCl_2_ 1, Na_2_-ATP 2, and Na-GTP 0.1 (pH adjusted to 7.2 with KOH) and had a resistance of 1.5–2.0 MΩ. All recordings were performed with an Axopatch-200B amplifier (Axon Instruments Inc., Foster City, CA, USA). Data collection and analysis were performed using PCLAMP 9 software.

### 2.6. Mouse Myocyte Mechanics, Ca Kinetics

To isolate RV and LV myocytes from 12-week-old anesthetized WT-PLN and R14del-PLN mice, the hearts were harvested and mounted in a Langendorff perfusion apparatus. The hearts were perfused with Ca-free Tyrode solution (described above) at 37 °C for 3 min. Subsequently, the hearts were perfused with Ca-free Tyrode solution, which contained the digestion enzyme liberase lendzyme I (0.25 mg/mL, Roche) until the hearts became flaccid (8–15 min). The RV and LV tissues were individually cut and shredded with two fine-tipped tweezers. Myocytes were released by pipette-dissociation followed by filtering through a 240 μm screen. The cell suspensions were transferred into 15 mL conical tubs, and the cells were allowed to settle for about 10–15 min. Then, the cell pellets were washed in 25, 100, 200 μM and 1 mM Ca-Tyrode. The cells were kept in 1 mM Ca-Tyrode buffer until the experiments were performed. For analysis, 10 uL of the cell suspension was transferred into a Plexiglas chamber containing 500 uL of 1.8 mM Ca-Tyrode buffer, which was positioned on the stage of an inverted epifluorescence microscope (Nikon Diaphot 200). Cell shortening and Ca-transients were measured at room temperature (24 °C) in RV and LV myocytes, stimulated at 0.5 Hz (+/−100 nM isoproterenol) [16]. For Ca transients, cells were loaded with Fura-2 (2 μM) and alternately excited at 340 and 380 nm by a Delta Scan dual-beam spectrophotofluorometer (Photon Technology Interbnational, Monmouth Junction, NJ, USA ) at baseline and upon rapid application of 10 mM caffeine. To examine SR Ca load and the contribution of Na/Ca exchanger during [Ca^2+^]i decline, the Fura2 loaded cells were stimulated at 0.5 Hz, and upon stabilization of twitches, 10 mM caffeine was rapidly applied and recording was continued for 20 s [16,17]. The caffeine-induced Ca transient amplitude was used as an indicator of SR Ca content. Decline of [Ca^2+^]i during a caffeine-induced Ca transient was attributed to Na/Ca exchange. In addition, myocyte mechanics were evaluated in the presence of 100 nmol/L isoproterenol at 0.5 Hz. Data were analyzed by Felix software (Photon Technology International, Birmingham, NJ, USA).

### 2.7. Measurement of Calcium Sparks

Ca sparks were recorded in quiescent LV and RV myocytes loaded with fluo-4 AM (10 µM; Molecular Probes, Eugene) and washed with Tyrode’s solution. Myocytes were superfused with Tyrode solution containing 1.8 mM CaCl_2_ and spontaneous Ca sparks were obtained in the absence or presence of 100 nmol/L isoproterenol [16,17]. Fluorescence images were recorded using a Zeiss LSM 710 inverted confocal microscope through a 40× water-immersion objective lens with excitation wavelength of 488 nm and the signals were measured at >515 nm with line-scan imaging at 3.07 ms intervals. Image processing and data analysis were performed using ImageJ PLUG IN (Wayne Rasband, National Institute of Health, USA).

### 2.8. Aftercontractions and CaMKII Inhibitor KN93 in Isolated Cardiomyocytes

Rod-shaped quiescent LV and RV myocytes were paced at 2 Hz in the presence of 1 µmol/L isoproterenol in 1.8 mmol/L Ca-Tyrode’s solution at room temperature. After stimulating the myocytes for a short time, pacing was stopped and spontaneous after-contractions were recorded within 5 s. In some experiments, myocytes were incubated with the calmodulin-dependent protein kinase II (CaMKII) inhibitor KN-93 (1 µmol/L, Sigma) or its analog KN92 (1 µmol/L, Sigma) as a control [16,17].

### 2.9. Immunofluorescence Staining and Quantitative Immunoblotting

Isolated RV and LV myocytes from WT-PLN and R14del-PLN hearts were plated in a laminin-coated Lab-Tek chamber slides (Thermo Scientific, Waltham, MA, USA). Myocytes were fixed with 1.2 mmol/L Ca-Tyrode solution containing 4% Paraformaldehyde (Electron Microscopy Sciences) for 30 min at room temperature and permeabilized with saline solution containing 0.1% Triton-X at 37 °C for 15 min. Non-specific binding was limited by 4 h incubation with blocking solution containing 2% goat serum in order to prevent the non-specific binding. Myocytes were stained with monoclonal anti-PLN (1:500; Thermo Fisher, Waltham, MA, USA) and polyclonal anti-SERCA2a (1:500; Badrilla, UK) overnight (4 °C), followed by incubation with Alexa flour 488 goat anti-rabbit (Licor, USA) and Alexa flour 594 goat anti-mouse (Licor, USA) secondary antibodies overnight at 4 °C. Images were acquired using a confocal microscope (Zeiss LSM 710 Live DUO). For quantitative immunoblotting, appropriate amount of protein from RV and LV tissue homogenates were separated in 12% SDS PAGE gels, blotted, and transferred to 0.1 to 0.2 μm nitrocellulose membrane. Subsequently, immunoblots were blocked using the Odyssey TBS blocking buffer (LI-COR Biosciences, Lincoln, NE, USA) at room temperature for 1 h. Next, blots were washed with TBS buffer 3 times for 10 min interval, followed by probing with primary antibodies. The primary antibodies were: PLN (1:1000; OriGene), SERCA2a (1:1000; Thermo Fisher), HRC (1:1000; Sigma), CSQ (1:1000; Affinity Bioreagent), HAX-1 (1:1000; BD Biosciences), and NCX (1:1000; Cell Signaling). For visualization of the proteins of interest, the blots were incubated with appropriate secondary antibodies (IRDye^®^ 680/800; Licor Biosciences; 1:5000), and images were obtained using the Odyssey^®^ CLx Imaging System.

### 2.10. RNA Sequencing Analysis

RNA sequencing was performed by the Cincinnati Children’s Hospital Medical Center DNA sequencing and genotyping core using an RNA polyA stranded library preparation [18]. CLC Genomics Workbench (v 20.0.2, Qiagen) was used to map sequenced reads to the mouse genome (GRCm38), perform principal component analysis, and identify differential expression changes (statistical significance threshold defined as an FDR-corrected *p*-value ≤ 0.05 and a fold change of ≥1.5).

### 2.11. Statistical Analysis

Twelve- to fourteen-week-old male mice were used. Data are expressed as mean ± standard error of the mean (SEM) for the number of mice, hearts or myocytes, and statistical analyses were performed by two-tailed unpaired *t*-test using GraphPad Prism version 8.4.3. Cardiomyocyte data are presented as individual data points, except for the frequency of Ca-sparks and the percent of cells showing after-contractions, which were presented as individual hearts [16,19]. All myocyte data were generated using LV and RV cells from the same mouse on any specific day.

A *p*-value of <0.05 was set for the level of significance. Significance is presented throughout as: * *p* ≤ 0.05, ** *p* ≤ 0.001, *** *p* ≤ 0.0001. For the differential gene expression studies, statistical significance was defined as an FDR-corrected *p*-value of ≤0.05 and a fold-change of ≥1.5.

## 3. Results

### 3.1. Ventricular Ectopy Originating in the Right Ventricle of Humanized Mice Harboring the R14del-PLN Mutation

**Figure 1:**
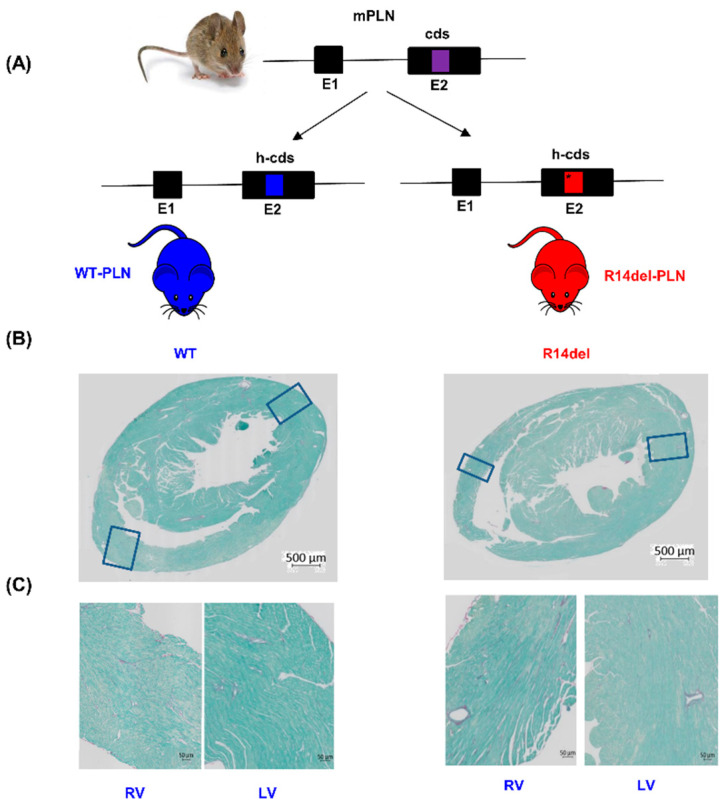
Generation of humanized knock-in R14del-PLN mice and cardiac histology. (**A**) Humanized wild-type (WT) and mutant (R14del) mouse models were generated by inserting the human wild-type and mutant PLN coding sequence (h-cds) into exon 2 of mouse PLN (mPLN; cds) gene-by-gene targeting methodology. (**B**) Representative cross-sections and (**C**) RV and LV sections from 3-month-old WT and R14del mice stained with Picro-Sirius Red and Fast Green solution. Magnification 20×; scale bar; 500 μm and 50 μm. N = 6 hearts for WT and N = 6 hearts for R14del.

To elucidate the early role of R14del-PLN in cardiac function, we generated and characterized knock-in mice expressing human WT or mutant PLN (Figure 1A) at a young age (12–14 weeks). Morphological and histological examination revealed no alterations (Figure 1Β,C).

**Figure 2:**
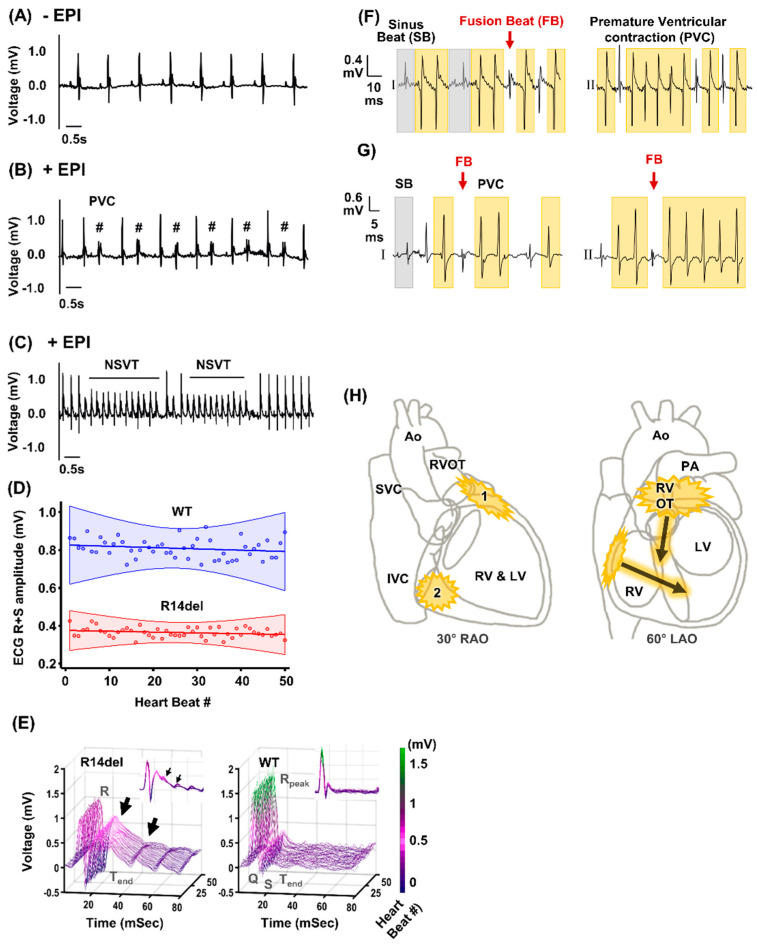
In vivo electrophysiological characteristics and putative source of the ventricular arrhythmias in R14del-PLN mutant mice. (**A**) ECG tracing under basal condition in R14del-PLN mice. (**B**,**C**) ECG tracings showing stress (caffeine and epinephrine) induced arrhythmias in the forms of premature ventricular complexes (PVCs, #), and non-sustained ventricular tachycardia (NSVT) in R14del-PLN mice (N = 5). (**D**) The mean R+S wave amplitudes with linear regression fit (solid line) and 95% confidence intervals (shaded) are shown for 50 consecutive heartbeats at a steady state for WT and R14del mice. (**E**) Representative ECG traces are plotted for 50 consecutive heartbeats at a steady state as a function of time and heartbeat number. The R14del mice (left panel) had reduced R wave amplitudes, prolonged QT and increased dispersion of repolarization (arrows) compared to wild type (WT). (**F**) Representative ECG tracings of PVC and VT beats from R14del-PLN animals are shown from leads I and II. The presence of fusion beats (red arrow) is diagnostic of PVC and non-sustained VT. Compared to the sinus beats (grey shaded areas), the morphology of the PVC and VT beats (yellow) suggests that these beats originated from or near the right ventricular outflow tract (RVOT). (**G**) Representative example of another common morphology of the PVC and VT beats from leads I and II for another R14del-PLN animal. The net QRS vector suggests these PVC and VT beats originated from the basolateral free wall of RV. (**H**) The two most common origin sites for PVC and VT in R14del-PLN mice are summarized in the 30° right anterior oblique (RAO) and 60° left anterior oblique (LAO) schematics of the heart (Ao = aorta; SVC = superior vena cava; IVC = inferior vena cava; PA = pulmonary artery; LV = left ventricle).

Since R14del-PLN is associated with arrhythmogenic RV cardiomyopathy in humans, we investigated whether there were any cardiac arrhythmias in vivo. The surface ECG was monitored before (baseline) and after caffeine and epinephrine administration (stress) (Figure 2A–C). At baseline, infrequent premature ventricular contractions (PVCs) were noted in 20% of WT or mutant mice. By contrast, stress caused a high frequency of PVCs in all (5/5) mutant mice, including ventricular bigeminy (Figure 2B). The stress-induced PVC burden, quantified over a 3-min period, was greater (10 ± 2 per min) in mutant than WT-PLN mice (3 ± 1 per min). Furthermore, episodes of non-sustained ventricular tachycardia (VT) occurred in all five mutant mice (Figure 2C) whereas, these were observed in only one of the five WTs. Despite similar heart rate responses to stress, the mutant exhibited prolongation of atrial and atrioventricular conduction, ventricular activation (QRS) and ventricular repolarization (JT) times (Table 1). These led to prolongation of the entire process from ventricular activation to repolarization (QT and QTc) (Table 1). The peak amplitude of ventricular depolarization was also reduced in R14del-PLN (Figure 2D). In addition, we noted significant heterogeneity in the beat-to-beat changes in ventricular repolarization (Figure 2E), which is known to be a strong independent predictor of VT and sudden cardiac death in humans [20,21].

The ECG findings on prolongation of PR and QT interval, as well as decreases in QRS peak voltage and R wave amplitude in mutant mice reflected similarities to presymptomatic R14del-PLN patients (Table 2). The prolongation of the PR interval observed in mice is a new finding in PLN patients, and it is in line with an ECG feature detection study using deep neural networks [22]. Interestingly, together with the R-wave attenuation, this was one of the early signs in R14del-PLN patients (Table 2), suggesting that the humanized mouse model recapitulates some of the patient characteristics. Although not found in the current patient cohort, common ECG findings in ARVC, such as prolonged terminal activation duration and epsilon waves, resemble the described QRS prolongation. These ECG abnormalities also seem to develop more frequently during exercise ECG testing, in both asymptomatic and symptomatic ARVC patients. A closer inspection of the distinct electrocardiogram QRS morphologies in mice (Figure 2F,G) suggested that the PVC and VT originated from at least two distinct regions, the basolateral RV free wall and the RV outflow tract (Figure 2H), similar to arrhythmogenic right ventricular cardiomyopathy (ARVC) patients [23].

### 3.2. R14del-PLN Associates with Prolongation of Action Potential in RV Myocytes

**Figure 3:**
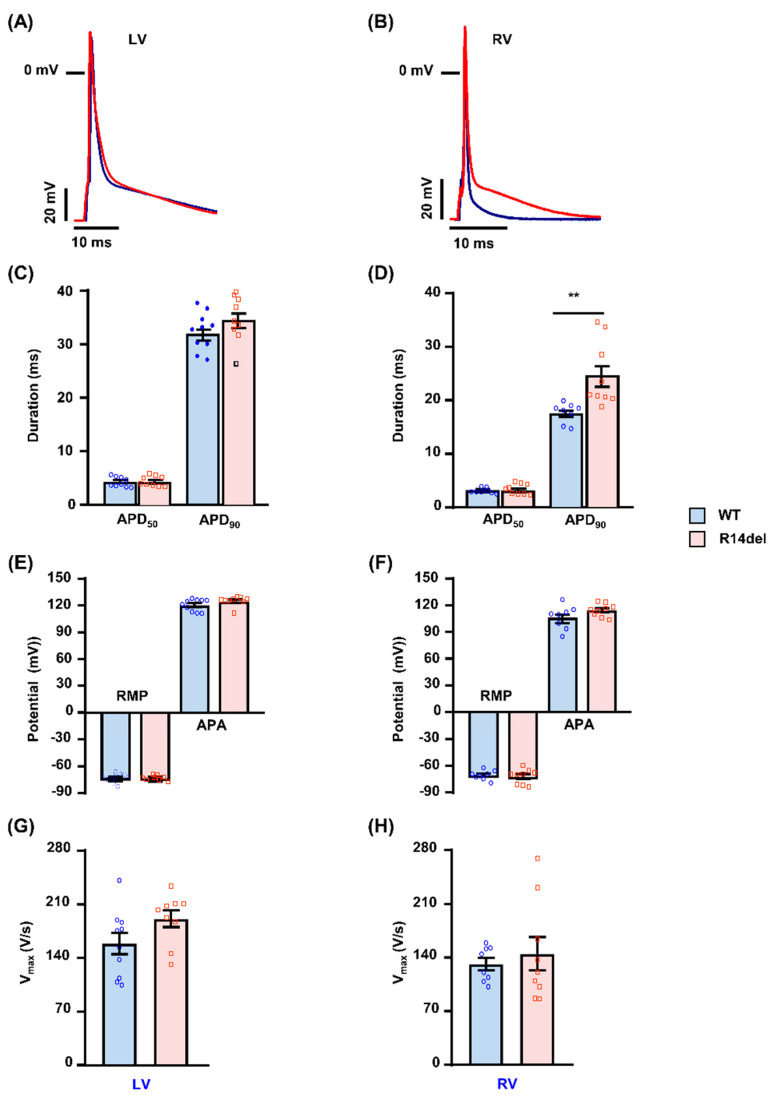
Action potential (AP) characteristics in cardiomyocytes from WT-PLN and R14del-PLN mice. APs were recorded in isolated LV and RV myocytes under basal conditions using whole-cell patch clamp recording under current clamp mode at room temperature. (**A**,**B**) Representative traces of action potential (AP) measured at 1 Hz. (**C**,**D**) Average AP duration (APD) at APD 50 and APD 90 repolarization. (**E**,**F**) Average resting membrane potential (RMP) and AP amplitude (APA). (**G**,**H**) Average maximal upstroke velocity (Vmax). n = 8 cells for LV and n = 10 cells for RV of WT (N = 3 hearts) and n = 9 cells for LV and n = 9 cells for RV of R14del-PLN (N = 3 hearts). Data are expressed as mean ± SEM of the total number of cells/group and statistical analyses were performed by Student’s unpaired *t*-test. ** *p* ≤ 0.001 vs. WT-PLN.

Since R14del is associated with arrhythmias, originating in the RV (Figure 3), we isolated RV and LV myocytes from WT-PLN and R14del-PLN mice and recorded the transmembrane AP waveforms under basal conditions (Figure 3A,B). As shown in Figure 3, no differences were noted in the resting membrane potential (RMP), AP amplitude (APA) and AP upstroke velocity (Vmax) from RV or LV myocytes between WT and R14del-PLN mice (Figure 3E–H). However, a prolonged AP duration was observed selectively in RV myocytes from R14del-PLN mice. The APD50 was not statistically prolonged, but the APD90 was significantly longer in the RV myocytes from R14del-PLN (Figure 3D). In contrast, such RV-sided APD prolongation was absent in LV myocytes (Figure 3C).

### 3.3. Calcium Kinetics and Contractile Parameters Are Depressed in RV Myocytes

**Figure 4:**
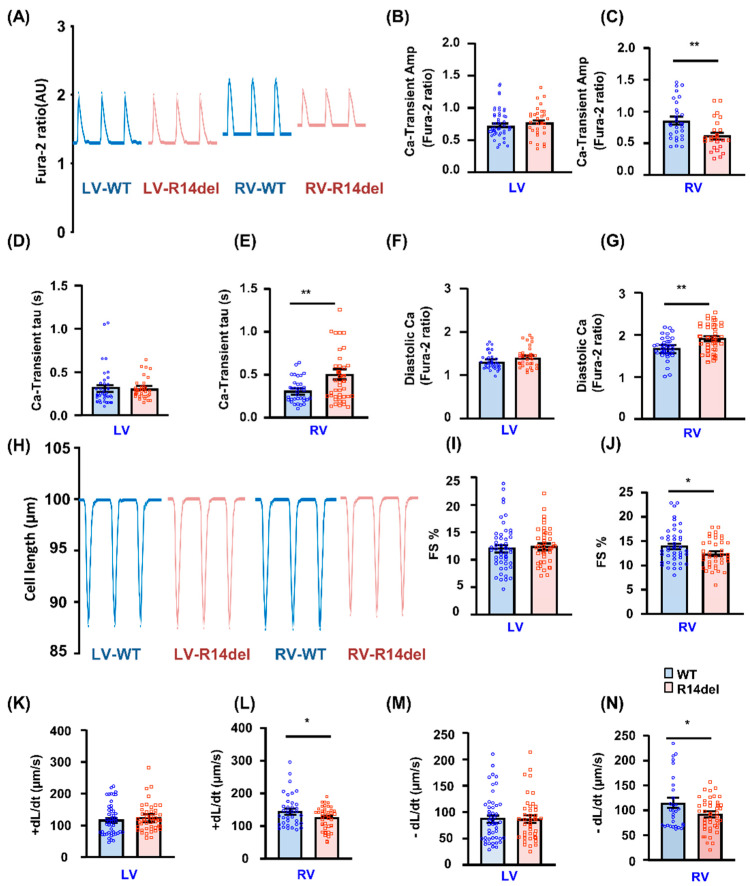
Ca-kinetic and contractile parameters in isolated cardiomyocytes. (**A**) Representative traces of Ca-transients; (**B**,**C**) Ca transient amplitude, indicated by fura-2 ratio (340:380 nm). (**D**,**E**) The relaxation time constant (Tau) of the Ca-transient decay. (**F**,**G**) Intracellular diastolic Ca levels in LV and RV myocytes. N = 36 LV and 35 RV cells for WT (N = 4 hearts); n = 34 LV and 41 RV cells for R14del-PLN (N = 4 hearts). (**H**) Representative cell shortening traces. (**I**–**N**) Contractile parameters: Fractional shortening (FS%) and maximum rates of contraction (+dL/dt) and re-lengthening (−dL/dt) in LV and RV myocytes at 0.5 Hz. N = 43 LV and 50 RV cells for WT (N = 4 hearts); n = 40 LV and 43 RV cells for R14del-PLN (N = 4 hearts. Data are expressed as mean ± SEM for the number of cells, and statistical analyses were performed by Student’s unpaired *t*-test. * *p* ≤ 0.05 vs. WT-PLN; ** *p* ≤ 0.001 vs. WT-PLN.

To gain further insights into intracellular Ca-cycling, we assessed Ca-transients in isolated cardiomyocytes by use of the Fura-2 AM fluorescence indicator (2 µM). In LV cells, the Ca-kinetic parameters were similar between WT and mutant hearts (Figure 4A,B,D,F). However, mutant PLN elicited decreases in the Ca-peak of the transient, prolongation of the time constant for Ca-decay (tau) and increases in diastolic Ca levels in RV myocytes (Figure 4A,C,E,G). Consistent with these findings, the fractional shortening (FS) and rates of contraction (+dl/dt) and relaxation (−dl/dt) were all significantly reduced only in RV mutant myocytes, while there were no parallel alterations in LV mutant cells (Figure 4H–N). To examine the effects of β-adrenergic agonists, cardiomyocytes were subjected to maximal isoproterenol (Iso; 100 nmol/L) stimulation. This resulted in enhanced contractility in both LV and RV myocytes, but the maximally stimulated parameters were lower in mutant RV cells (Figure S1A–F). Thus, R14del-PLN elicits depressed RV myocyte Ca-kinetics and contractile parameters, which may not be fully relieved even upon iso-stimulation.

**Figure 5:**
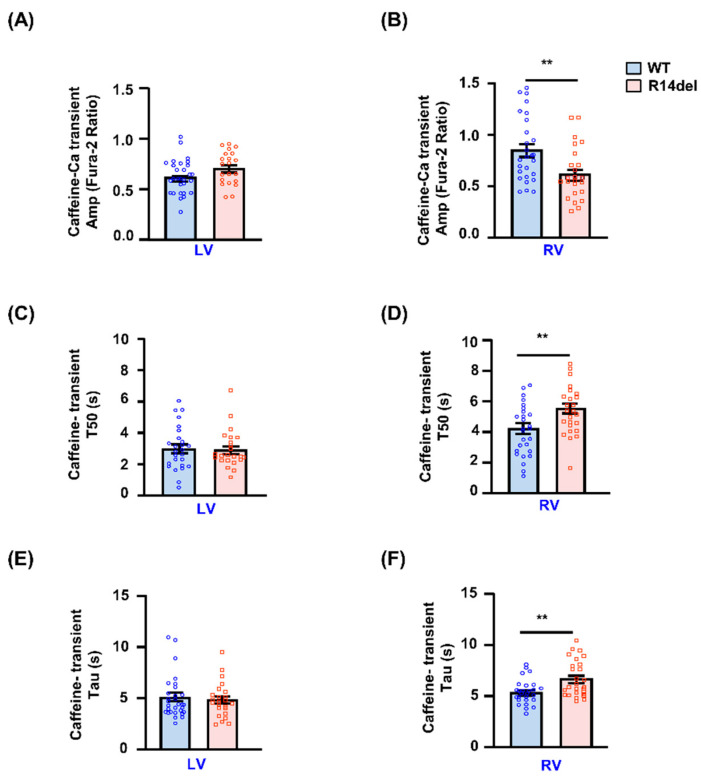
Sarcoplasmic reticulum Ca-load and NCX activity. SR Ca load and NCX function were assessed under caffeine (20 mM/L) application. Caffeine transients were measured in LV and RV cardiomyocytes loaded with the Fura-2 AM fluorescence indicator (2 µM). (**A**,**B**) Amplitude of caffeine-induced Ca transients showing SR Ca load in LV and RV myocytes, as indicated by the fura-2 ratio (340:380 nm). (**C**,**D**) T50: the time constant for 50% decay of the caffeine-induced Ca transient. (**E**,**F**) Tau of caffeine-induced Ca transient decay. n = 27 LV and 28 RV cells for WT (N = 3 hearts); n = 27 LV and 29 RV cells for R14del-PLN (N = 4 hearts). Data are expressed as mean ± SEM for the number of cells, and statistical analyses were performed by Student’s unpaired *t*-test. ** *p* ≤ 0.001 vs. WT-PLN.

The effects of R14del-PLN on intracellular Ca-cycling prompted further studies on the influence of this mutant on SR Ca-content. There were no significant differences in the amplitude of caffeine-induced Ca-release or the decay time of this transient (T50 or Tau) between LV myocytes from mutant and WTs (Figure 5A,C,E). However, the caffeine-induced Ca^2+^ transient peak was reduced in mutant RV myocytes compared to WT-PLN cells, which indicates lower SR Ca-content (Figure 5B). In addition, the time to 50% decay (T50) and the time constant of cytosolic Ca-decline (Tau) during the caffeine-induced Ca-transient were increased in RV myocytes (Figure 5D,F). This suggests that the activity of the sodium/calcium exchanger (NCX) regulating Ca extrusion was decreased. Furthermore, to determine how much Ca is released at each twitch relative to the total amount of Ca stored, we examined the fractional release by normalizing the electrically evoked Ca-transient to the caffeine-evoked Ca-transient. Fractional release was not different between WT and mutant myocytes from either the LV or the RV (data not shown).

### 3.4. Calcium Sparks and After-Contractions Are Increased in RV Myocytes from R14del-PLN Hearts

**Figure 6:**
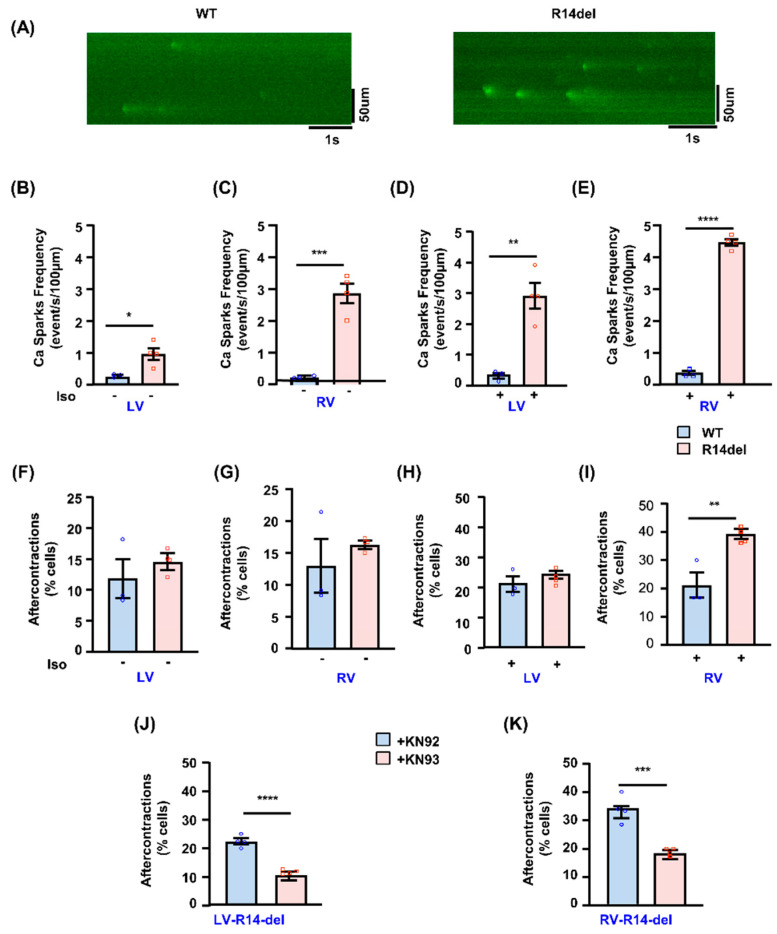
Ca sparks and aftercontractions in R14de-PLN cardiomyocytes. Ca spark characteristics in isolated LV and RV myocytes. (**A**) Representative line-scan images of Ca sparks in RV myocytes in the presence of 100 nmol/L isoproterenol. (**B**,**C**) Mean data of Ca spark frequency under basal conditions. (**D**,**E**) Mean data of Ca spark frequency in presence of 100 nmol/L isoproterenol (Iso). n = 30 LV and 30 RV cells for WT (N = 3 hearts); n = 30 LV and 30 RV cells for R14del-PLN (N = 4 hearts). (**F**–**I**) Aftercontractions were assessed in LV and RV myocytes under field stimulation of 2 Hz and in the absence (**F**,**G**) or presence (**H**,**I**) of 1 μmol/L isoproterenol. n = 49 LV and 52 cells RV cells for WT (N = 3 hearts); n = 65 LV and 58 RV cells for R14del-PLN (N = 4 hearts). (**J**,**K**) Assessment of aftercontractions in presence of CaMKII inhibitor KN93 (1 µmol/L) or its analog KN92 (1 µmol/L) as control. n = 57 LV and 54 RV cells for WT (N = 4 hearts and n = 54 LV and 63 RV cells for R14del-PLN (N = 4 hearts). Data are expressed as mean ± SEM, and statistical analyses were performed by Student’s unpaired *t*-test. * *p* ≤ 0.05 vs. WT-PLN; ** *p* ≤ 0.001 vs. WT-PLN; *** *p* ≤0.0001 vs. KN92; **** *p* ≤ 0.00001 vs. KN92.

The effects of R14del-PLN on RV cardiomyocyte diastolic calcium prompted further studies on its influence on the frequency of Ca-sparks (Figure 6A), which may serve as a molecular trigger to arrhythmia [16]. Indeed, the frequency of Ca sparks was significantly higher in mutant RV myocytes compared to LV (Figure 6B,C), indicating increased SR Ca-leak and arrhythmogenic propensity. Isoproterenol stimulation increased the Ca-spark frequency, and the enhancement was greater in RV cells (Figure 6D,E). Indeed, RV mutant cardiomyocytes exhibited significantly higher spontaneous aftercontractions (2-Hz + 1 µmol/L isoproterenol) compared to WT counterparts (Figure 6H,I), while this was not observed in LV mutant cardiomyocytes (Figure 6F,G). Interestingly, the inclusion of KN93 [16], the selective inhibitor of CaM kinase II, abrogated the increased after-contractions in mutant RV cells (Figure 6K), indicating that aberrant SR Ca release was associated with increased CaMKII activity, contributing to ACs. Control studies used KN92, the inactive analog of KN93 (Figure 6J,K).

### 3.5. Calcium Handling Protein Levels

**Figure 7:**
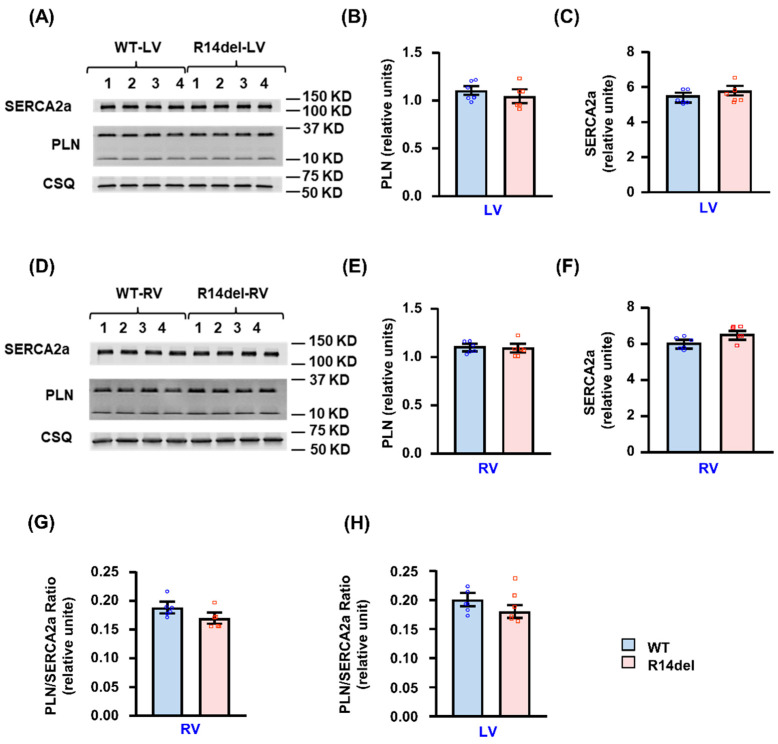
Assessment of PLN levels in LV and RV. (**A**) Representative immunoblots of PLN and SERCA2a protein levels in LV from WT-PLN and R14del-PLN. (**B**) Quantitative assessment of PLN protein levels in LV. (**C**) Quantitative assessment of SERCA2a protein levels in LV. Calsequestrin (CSQ) was used as a loading control. N = 6 LV samples from WT and N = 6 LV samples from R14del-PLN mice. (**D**) Representative immunoblots of PLN and SERCA2a protein levels in RV from WT-PLN and R14del-PLN. (**E**) Quantitative assessment of PLN protein levels in RV. (**F**) Quantitative assessment of SERCA2a protein levels in RV. Calsequestrin (CSQ) was used as a loading control. N = 6 RV samples from WT and N = 6 RV samples from R14del-PLN mice. (**G**,**H**) The PLN/SERCA2a ratio in RV and LV samples. Twelve- to fourteen-week-old male mice were used. Data are expressed as mean ± SEM for the number of hearts and statistical analyses were performed by two-tailed unpaired *t*-test. A *p*-value of <0.05 was considered statistically significant.

The observed RV-specific alterations in Ca-kinetic and contractile parameters by R14del-PLN prompted evaluation of the expression levels of PLN and other key SR Ca-cycling proteins in RV and LV compartments. Quantitative immunoblotting indicated that the PLN levels were similar between WT or R14del in either the LV or RV compartments (Figure 7A,B,D,E). In addition, the SERCA2a levels were also similar (Figure 7A,C,D,F). Thus, there were no differences in the PLN/SERCA2a relative ratio between LV or RV in either WT or R14del-PLN hearts (Figure 7G,H). Furthermore, this ratio was evaluated using transcriptomic analyses, and it was similar between LV and RV in either WT or mutant hearts (0.6 ± 0.02 in RV and 0.6 ± 0.03 in LV of WTs; 0.63 ± 0.01 in RV and 0.58 ± 0.04 in LV of mutants).

**Figure 8:**
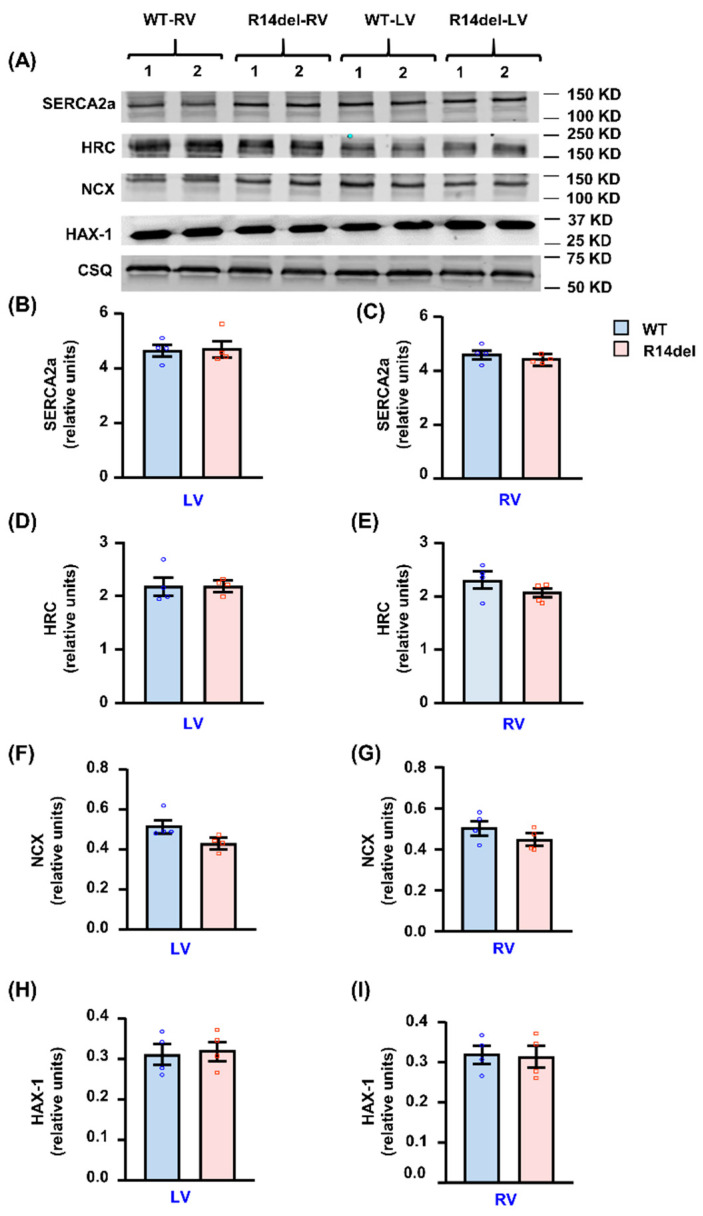
Assessment of SR calcium cycling protein levels in RV and LV of WT and R14del-PLN mice. (**A**) Representative immunoblots of Ca cycling protein levels in WT and R14del ventricles. (**B**,**C**) Quantitative assessment of SERCA2a protein levels. (**D**,**E**) Quantitative assessment of HRC protein levels. (**F**,**G**) Quantitative assessment of NCX protein levels. (**H**,**I**) Quantitative assessment of HAX-1 protein levels. Calsequestrin (CSQ) was used as a loading control. N = 4 RV and LV samples from WT and N = 4 RV and LV samples from R14del-PLN mice. Twelve- to fourteen-week-old male mice were used. Data are expressed as mean ± SEM for the number of hearts and statistical analyses were performed by two-tailed unpaired *t*-test. A *p*-value of <0.05 was considered statistically significant.

Analysis of other Ca-cycling proteins indicated no alterations in the levels of HAX-1, HRC and NCX by R14del-PLN in either RV or LV compartments (Figure 8). Further immunofluorescence examination of cardiomyocytes revealed that both WT and R14del-PLN co-localized with SERCA2a in LV and RV myocytes (Figure S2A,B) although a few cells showed a granulated pattern for mutant PLN (Figure S2C). This observed cell heterogeneity and PLN aggresomes are consistent with findings in R14del-PLN patients’ hearts [5].

### 3.6. Gene Expression Profiles

**Figure 9:**
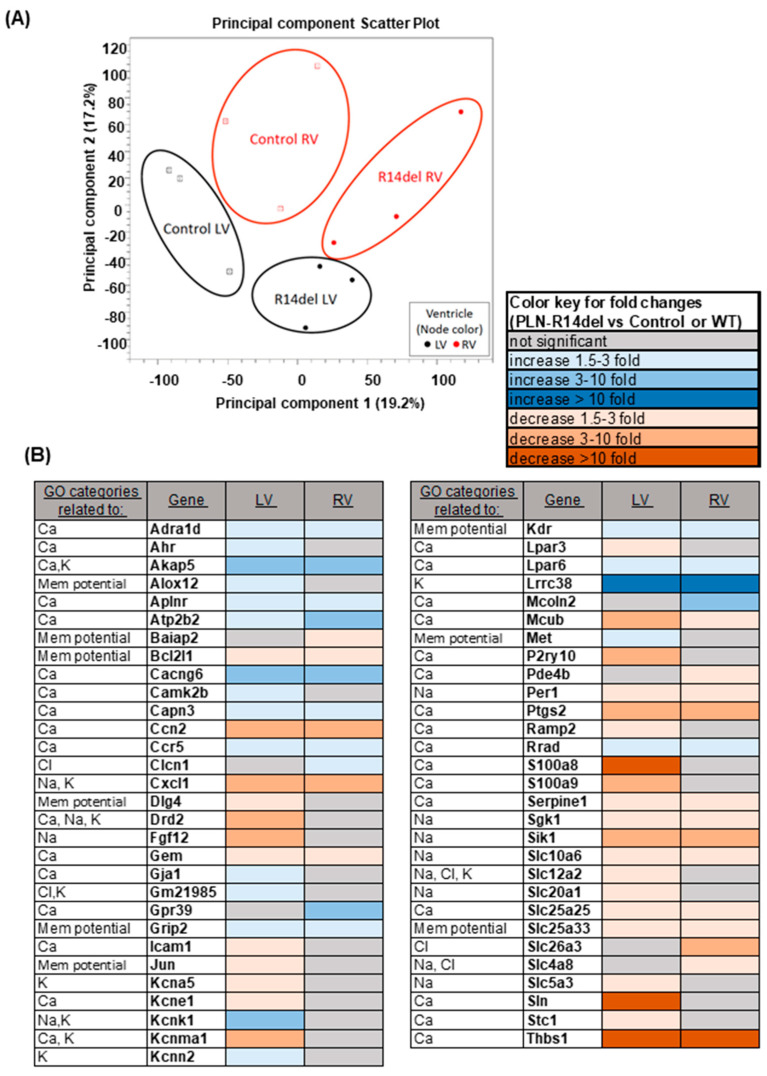
Identification of R14del-PLN Mutation Changes in Electrophysiological and Ca Cycling Transcript Levels. (**A**) The principal component analysis is based on normalized expression values for RNA seq data for the RV and LV of R14-PLN and Control or WT-PLN mice (N = 3). (**B**) Significantly changed Gene Ontology categories (*p* ≤ 0.05) related to membrane potential and ion homeostasis changes, based on the analysis of the significantly changed genes (FDR-corrected *p*-value ≤ 0.05 and fold ≥ |1.5|) detected between PLN-R14del LV and WT (Control) LV, or PLN-R14del RV and WT (Control) RV. The table includes the gene symbol and level of fold change in the LV and RV, based on the provided color key.

To obtain a further unbiased view of potential pathway changes associated with the RV-specific effects of R14del-PLN, global gene expression profiling was performed using next-generation sequencing in LV and RV samples from WT and mutant hearts. Principal component analysis showed independent clustering of each sample group, indicating a unique gene expression signature within each group (Figure 9A). Collectively, the R14del-PLN mutation was associated with significant expression changes of 515 genes in the LV and 306 genes in the RV, with 233 being common between two ventricles. Gene Ontology (GO) analysis revealed significant changes in genes related to Ca-cycling, membrane potential and ion transport (Figure 9B). Among them, sarcolipin (Sln), which regulates SERCA2a activity in a manner analogous to PLN, was significantly downregulated only in LV. This observed decrease in Sln was also confirmed at the protein level (data not shown). In addition, sarcolipin was also under-expressed in LV samples from human R14del-PLN patients (unpublished data by Drs Pei, Harakalova and Folkert). Other LV-specific under expressed genes included the Ca-binding proteins S100a8/S100a9, the purinergic receptor P2ry10, which may be involved in calcium release from intracellular stores, and the calcium-activated potassium channel Kcnma1, which contributes to hyperpolarization of the cell membrane. These alterations in LV may serve as potential compensation to normalize the inhibited SR Ca-cycling by mutant PLN. Parallel observations in RV revealed underexpression of solute carrier family 26 member 3 (Slc26a3), which regulates intracellular pH and thus Ca, and overexpression of mucolipin 2 (Mcoln2), which belongs to the transient receptor potential channel family and acts as a Ca-permeable cation channel. These alterations in RV may contribute to impaired Ca-cycling, especially since Mcoln2 has been linked to arrhythmias in GWAS data from the GWASdb SNP-Phenotype Associations dataset [24]. Notably, there were no alterations in myoregulin, endoregulin, any other-regulin or DWORF, the newly identified SERCA2a regulatory proteins [25].

Analysis of other significantly altered GO categories included ER stress and UPR pathways, which may be associated with early responses in mutant hearts due to aberrant SR Ca-handling. We observed downregulation of several genes, which were common between LV and RV (Figure S3). The most prominent ones were thrombospondin-1 (Thbs1), which is a Ca-binding protein that may control ER stress; several heat shock proteins (Hspa1a, Hspa1b, Dnaja1 and Dnajb1), which act as molecular chaperones involved in protein quality control and correct folding; and glutathione specific gamma-glutamylcyclotransferase 1 (Chac1), which is a regulator of the unfolded protein response (UPR) pathway, downstream of Atf3. Additional alterations, associated with apoptosis/cell death pathways (Figure S4) in both ventricles, included downregulation of the anti-apoptotic proteins Bcl-2-like 1 (Bcl2l1), Pim-3 proto-oncogene serine/threonine kinase (Pim3), TIMP Metallopeptidase Inhibitor 3 (TIMP3) and dual specificity phosphatase 1 (Dusp1). Collectively, the observed decreases in ER-stress, UPR and apoptosis-related transcripts may contribute to early impairment of protein quality control and reduced protection in the R14del-PLN hearts, even though there is no apparent remodeling or a pathological phenotype at this early age.

Quantitative PCR was also performed to validate the findings for some of the genes identified in the RNA sequencing (Slc106, Lpar6, Serpine, Adra1a, Thbs1 and Rrad) and each gene showed the same trend in qPCR as in RNA sequencing although the values were not significant for all the genes. In future studies, the observed alterations at the transcriptional level presented above will need to be validated at the protein level and the involvement verified of specific protein players and pathways in the R14del-PLN phenotype.

## 4. Discussion

This is the first study to show that an inherited mutation in a Ca-regulatory protein that originates in RV-specific Ca-defects, leading to catecholaminergic malignant arrhythmia at a young age. The R14del-PLN mutation causes impaired SR Ca-sequestration and increased diastolic Ca levels, which trigger Ca-leak and arrhythmogenic action potential changes, ensuing ectopy that originates in RV. A major new finding is that the distinct ECG features in humanized models, including prolonged PR interval and attenuated R-waves, are remarkably similar to those observed in pre-symptomatic R14del-PLN patients at high risk for VT and sudden cardiac death [22,26]. In most genetically associated cardiomyopathy cases, ventricular arrhythmias appear to occur through progression to advanced heart failure. However, our current work suggests that the R14del-PLN mutation may increase the propensity to arrhythmia even at an early age in mice, similar to the increased risk of malignant VT in young patients [5]. As disease penetrance is incomplete and age-dependent, follow-up frequency and timing of therapeutic interventions are still major challenges in R14del-PLN patients [5]. The ECG changes and ventricular arrhythmias described during isoproterenol testing in these young mice may have clinical utility for the early identification of high-risk patients in asymptomatic carriers. While previous studies have shown the utility of isoproterenol testing for early stratification of ARVC patients, this study is the first to indicate its potential value specifically in R14del-PLN patients [26].

### 4.1. Ventricular Tachycardia and Its Origin in RV

The delayed ventricular activation and conduction times predispose the R14Rdel-PLN mice to reentrant ventricular tachycardia (VT). In addition, the prolonged and heterogenous ventricular repolarization predispose the mutant mice to triggered PVCs and VT [20]. Importantly, the PVCs and VT appeared to originate from the RV, consistent with delayed Ca-transient decay/relaxation and action potential prolongation in these myocytes. The triggered action potential in the RV myocytes (“source”) would produce a voltage gradient with the other end of the heart (“sink”), and then the current flows from source to sink to depolarize and trigger action potential in the cells downstream. Our data suggest that multiple factors alter the source and sink properties in R14del-PLN hearts, thereby dynamically regulating intercellular impulse propagation. The source is affected by the slower rate, reduced amplitude and prolonged duration of ventricular activation. The sink is affected by reductions in ventricular repolarization reserve and liminal length [27], the amount of tissue that must be depolarized above the threshold for action potential propagation. Notably, the reduced repolarization reserve exaggerates the dispersion of ventricular repolarization, thereby promoting ventricular arrhythmias [20]. In addition, our studies employing ECG multi-lead vector analysis suggest that the PVCs in the R14del-PLN mice originate from at least two distinct regions in the RV, the basolateral free wall and outflow tract (Figure 1). These findings are similar to our prior observations in ARVC patients [23]. Based on the remodeling of the source and sink described above, prolongation of ventricular repolarization in RV, in the setting of normal LV repolarization, would allow the triggered afterdepolarizations in RV to initiate PVCs that depolarize LV tissue (which already have recovered) to initiate reentrant VT. These processes likely account for the increased propensity of R14del-PLN mice to both triggered and reentrant VT under stress conditions [20,28].

Although stress ECG studies in human PLN patients are scarce, studies in ARVC patients showed that polymorphic PVCs, non-sustained VTs and sustained VTs originating from the RV are common during isoproterenol infusion and exercise testing [29]. Moreover, one study in PLN patients with ARVC phenotype showed that right ventricular VTs have incidence rates of up to 79% [30]. In PLN patients with a DCM phenotype, ventricular ectopy and arrhythmias are also common during exercise and exercise testing, but no studies have described the origin of these arrhythmias in detail [5,26].

### 4.2. Right Ventricular Specificity of R14del-PLN

Cellular characterization studies have mainly been performed in LV myocytes, and parallel studies in RV cells are overall limiting. Thus, potential compartment-specific differences may have gone unnoticed regarding the function of specific genes. Interestingly, R14del-PLN appeared to exert RV-specific depressive effects associated with prolonged Ca-transient decay, decreased SR Ca-load, elevated diastolic calcium levels and increased Ca-spark frequency, as well as aftercontractions. Potential explanations could include differences in PLN expression levels, the PLN/SERCA2a ratio or other key Ca-cycling proteins. However, all these were similar between LV and RV in WT-PLN or R14del-PLN hearts. Another possibility is the inherent differences between the two cardiac compartments including wall thickness, preload, ionic currents, structural organization and compensatory mechanisms. Indeed, NCX activity was decreased in RV myocytes, which may constitute an important compensatory mechanism in the face of inhibited SERCA2a activity to increase SR Ca-load but may also contribute to increased diastolic Ca [31]. Other RV-specific transcriptional changes, which may contribute to the observed phenotype, included underexpression of Slc26a3, which controls intracellular pH and may impact Ca-cycling, and upregulation of Mcoln2, which may participate in Ca-overload and potential arrhythmia [32,33]. Interestingly, Sln was significantly under-expressed only in LV of mutant mice, and this was confirmed in human carriers. Sln is an inhibitor of SERCA2a, similar to PLN, and decreases in its expression may partially offset the increased inhibitory effects of R14del in LV, preventing aberrant Ca-cycling. Although Sln is predominantly expressed in atria, its ablation significantly increased the Ca-affinity of SERCA2a in the ventricle [34], and its downregulation by AAV treatment improved cardiac function in a Duchenne muscular dystrophy model [35]. Overall, our findings on the RV-specific impact by R14del-PLN point to the importance of this compartment in driving the in vivo phenotype on increased propensity to arrhythmias, originating in RV.

### 4.3. Decreases in ER Stress and Apoptosis Pathways

Since alterations in Ca-cycling may affect cellular pathways involved in disease progression, we examined the expression of ER stress and UPR associated genes that may impact remodeling at an early stage. There was significant under-expression of several genes, including Thbs1, which is involved in ER stress responses and whose decreases may activate apoptotic mechanisms [36], and Hsp70 (Hspa1a and Hspa1b), which acts as molecular chaperone and whose under-expression may associated with incorrect folding and compromised protein degradation. In addition, Chac1, which is a regulator of the UPR pathway, downstream of Atf3 (transcriptional activator of UPR target genes) was significantly downregulated. Other alterations associated with apoptosis/cell death pathways were reflected in the under-expression of Bcl2l1, Dusp1 and Pim3, which have important anti-apoptotic roles, and Timp3, which inhibits the activity of metalloproteases. As a whole, the observed alterations suggest that the UPR, ER stress and apoptosis-related genes may underlie early pathology, which is eventually associated with protein aggregation, observed in R14del-PLN cardiomyopathy [5], and compromised cardioprotection mechanisms. However, beyond the changes observed at the transcriptional level, there may be post-transcriptional and/or post-translational modifications, introducing additional layers of changes that could contribute to R14del-PLN pathogenesis.

### 4.4. Study Limitations

There are several limitations associated with the current findings: (a) other factors, including environmental, genetic or epigenetic effects, may also contribute to the R14del-PLN phenotype; (b) findings in mouse hearts may not reflect similar alterations in large animal or human hearts as there are species differences, including heart rate, myosin heavy chain isoforms, Ca-cycling properties and ion currents; (c) the studies in isolated cardiomyocytes may be potentially biased by selection of the healthiest cells that survive the isolation procedure; (d) the detrimental effects of R14del-PLN may be mediated by additional pathways besides impaired SR Ca-cycling; and (e) the effects of gender were not addressed in the current study, which was conducted only in male mice. Thus, translation of the current findings and their implications for human females remain limited. Finally, the specific involvement of RV in this non-desmosomal model of ARVC is not entirely clear, although we speculate that inherent differences between LV and RV and compensatory mechanisms may account for these observations. Overall, the current study has provided significant insights into early cellular mechanisms associated with the naturally occurring R14del genetic variant.

## 5. Conclusions

Our findings in young humanized R14del-PLN mice indicate increased propensity to aftercontractions under stress conditions, resulting in delayed ventricular activation, prolonged repolarization and ventricular tachyarrhythmia, which appeared to originate from the right ventricle in vivo. At the cellular level, the mechanisms underlying arrhythmia involved increased inhibition of SR Ca-transport, increased SR Ca-leak and aftercontractions, as well as prolongation of action potential duration. Interestingly, the observed Ca-defects were specific to right ventricular mutant myocytes and reveal that RV-specific Ca-impairment is an early sign associated with R14del-PLN mutation. In addition, they are in agreement with findings in classic ARVC, showing primarily malignant ventricular arrhythmias during isoproterenol testing in the early stages of the disease. This mutant Ca-regulatory protein elicited characteristics similar to those of desmosomal gene mutations in intact animals. Furthermore, the observed ECG changes in young mice were similar to the pre-symptomatic R14del-PLN probands at an early age [3,5]. These findings highlight the importance of understanding early cellular defects that associate with R14del-PLN, providing the opportunity for targeted therapy. They also point to the paramount importance of genetic and cardiac screening for this rare exome variant, R14del-PLN, to prevent the increased risk for arrhythmic events at an early age. Early detection of high-risk mutation carriers, before the onset of symptoms, would allow for increased monitoring or interventions to prevent life-threatening ventricular arrhythmias.

## Figures and Tables

**Table 1:** Cardiac electrocardiographic assessment in WT-PLN and R14del-PLN mice under in vivo stress conditions.

	WT-PLN	R14del-PLN
Heart rate (bpm)	498 ± 28	483 ± 15
PR interval (ms)	41.6 ± 1.7	50.3 ± 1.9 *
QRS interval (ms)	9.6 ± 0.2	10.8 ± 0.3 *
Peak QRS voltage (mV)	1.53 ± 0.05	0.42 ± 0.09 **
JT interval (ms)	12.2 ± 2.3	49.2 ± 2.2 **
QT interval (ms)	21.8 ± 2.2	60.0 ± 2.5 **
QTc interval (ms)	19.7 ± 1.6	53.7 ± 1.9 **
R wave amplitude (mV)	1.29 ± 0.09	0.86 ± 0.09 *

Mice were anesthetized and monitored by surface ECG recordings after caffeine (120 mg/KG) and epinephrine (2 mg/Kg) application. Data are expressed as mean ± SEM for number of mice (N = 4 WT and 3 R14del-PLN). Statistical analyses were performed by unpaired *t*-test. * *p* ≤ 0.05 vs. WT-PLN, ** *p* ≤ 0.001 vs. WT-PLN.

**Table 2:** ECG characteristics of pre-symptomatic and symptomatic R14del-PLN mutation carriers and their age and sex-matched controls.

	Human		
	Controls	Pre-Symptomatic R14del-PLN	Symptomatic R14del-PLN
N	272	15	53
Age, years	48 ± 0.8	46 ± 3.3	48 ± 1.7
Heart rate, bpm	71 ± 0.9	62 ± 3.5 *	74 ± 2.5
PR interval, ms	153 ± 1.3	162 ± 7.6	180 ± 7.1 **
QRS interval, ms	95 ± 1.0	89 ± 2.2 *	98 ± 3.5 *
Peak QRS voltage, mV	1.1 ± 0.02	0.7 ± 0.08 **	0.6 ± 0.04 **
QT interval, ms	387 ± 1.9	419 ± 11.9 **	398 ± 6.5 *
QTc interval, ms	418 ± 1.6	417 ± 5.5	433 ± 6.0 **
R wave amplitude, mV	1.0 ± 0.02	0.6 ± 0.07 **	0.4 ± 0.04 **

For every patient, four controls were sampled from the same UMCU ECG database using propensity score matching for age and sex. Asymptomatic R14del-PLNpatients had no cardiac symptoms, no history of (non-)sustained ventricular tachycardia, premature ventricular complex burden of <500 beats per 24 h and left ventricular ejection fraction >45%. Asymptomatic patients who became symptomatic later were categorized as pre-symptomatic. Only ECGs of pre-symptomatic (n = 15) and symptomatic patients (n = 53) were analyzed. Data are expressed as mean ± SEM for the number of human subjects. Statistical analyses were performed by one-way ANOVA. * *p* ≤ 0.05, ** *p* ≤ 0.001.

## Data Availability

All RNA-seq data are available in the NCBI Gene Expression Omnibus (https://www.ncbi.nlm.nih.gov/geo/) (GSE173684).

## Supplementary Materials

The following are available online at https://www.mdpi.com/article/10.3390/jpm11060502/s1, Figure S1: Iso-stimulated contractile parameters; Figure S2: Assessment of PLN and SERCA2a co-localization in isolated ventricular myocytes of WT and R14del-PLN mouse hearts using immunofluorescence staining and confocal microscopy; Figure S3: ER and UPR gene alterations; Figure S4: Apoptosis gene alterations.
