# Supplementary material for: Impaired Right Ventricular Calcium Cycling Is an Early Risk Factor in R14del-Phospholamban Arrhythmias
Source: J Pers Med. 2021 Jun 3;11(6):502. doi: 10.3390/jpm11060502 (PMC8226909; doi:10.3390/jpm11060502)
Supplement: Supplementary file 1 [file jpm-11-00502-s001.zip › jpm-1233271-supplementary.pptx]

## Slide 1
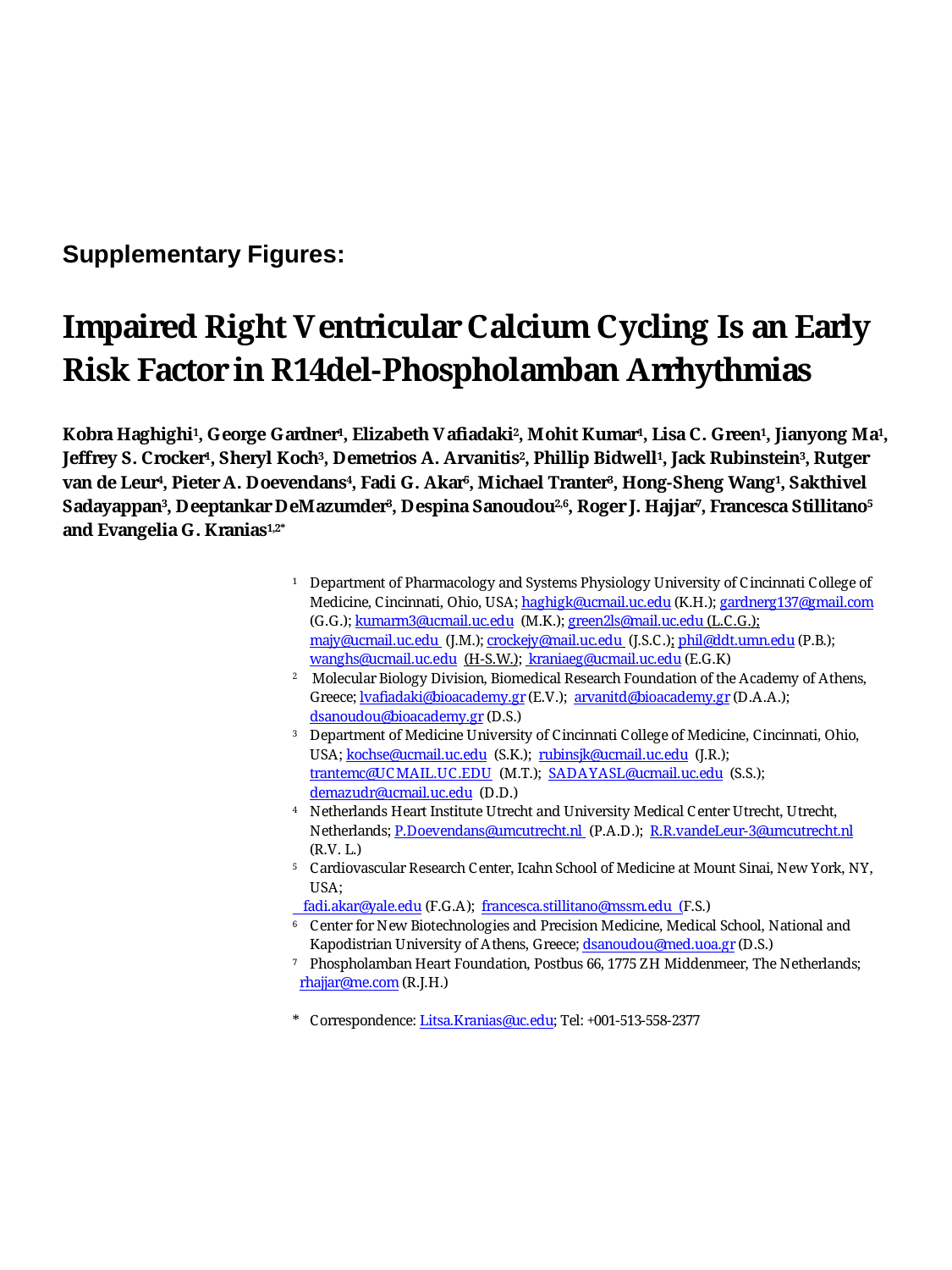

## Slide 2
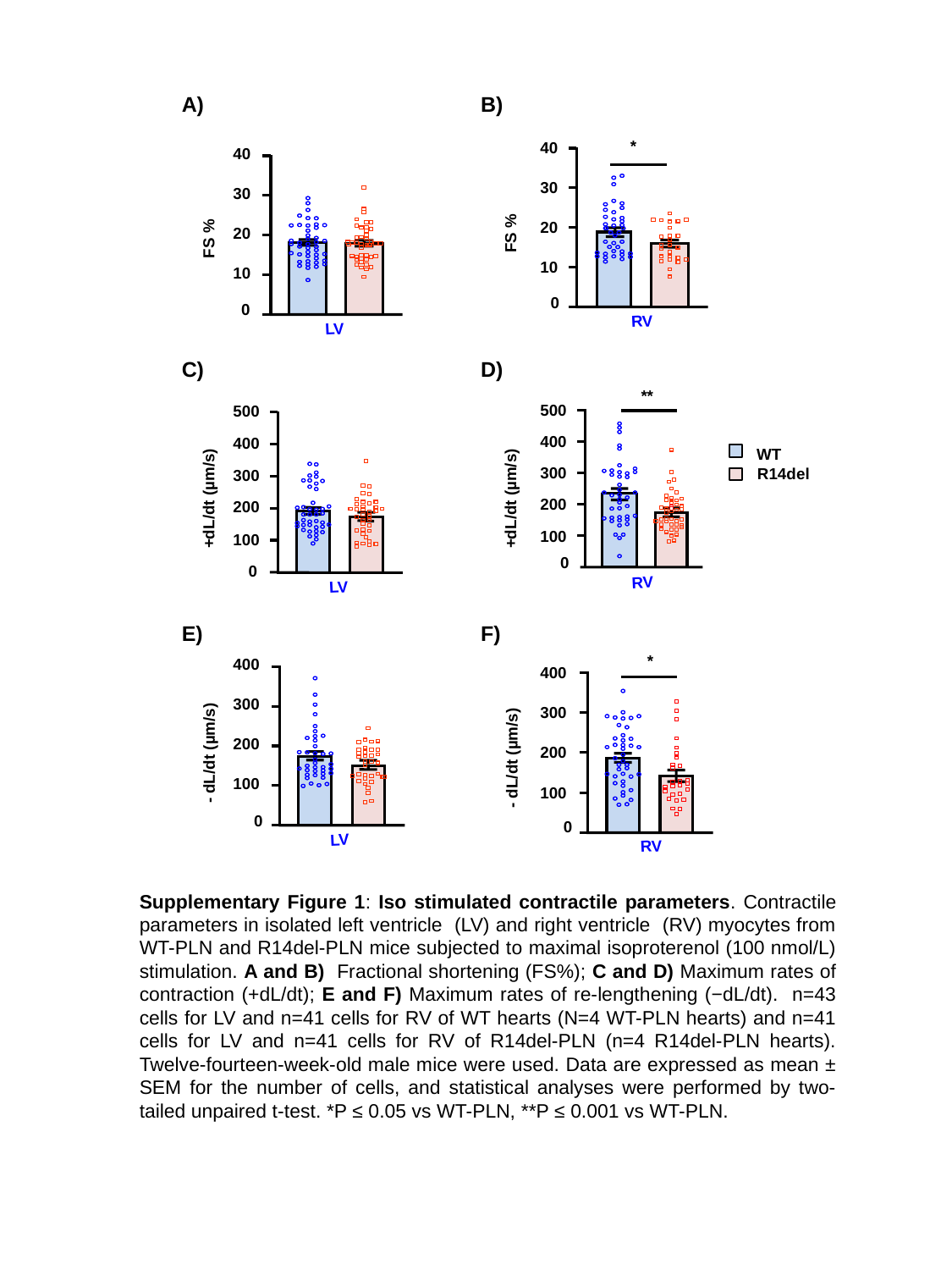

A)
B)
*
40
30
FS %
20
10
0
40
30
FS %
20
10
0
RV
LV
C)
D)
**
500
400
300
+dL/dt (µm/s)
200
100
0
500
400
300
+dL/dt (µm/s)
200
100
0
WT
R14del
RV
LV
E)
F)
*
400
300
- dL/dt (µm/s)
200
100
0
400
300
- dL/dt (µm/s)
200
100
0
LV
RV
Supplementary Figure 1: Iso stimulated contractile parameters. Contractile parameters in isolated left ventricle (LV) and right ventricle (RV) myocytes from WT-PLN and R14del-PLN mice subjected to maximal isoproterenol (100 nmol/L) stimulation. A and B) Fractional shortening (FS%); C and D) Maximum rates of contraction (+dL/dt); E and F) Maximum rates of re-lengthening (−dL/dt). n=43 cells for LV and n=41 cells for RV of WT hearts (N=4 WT-PLN hearts) and n=41 cells for LV and n=41 cells for RV of R14del-PLN (n=4 R14del-PLN hearts). Twelve-fourteen-week-old male mice were used. Data are expressed as mean ± SEM for the number of cells, and statistical analyses were performed by two-tailed unpaired t-test. *P ≤ 0.05 vs WT-PLN, **P ≤ 0.001 vs WT-PLN.

## Slide 3
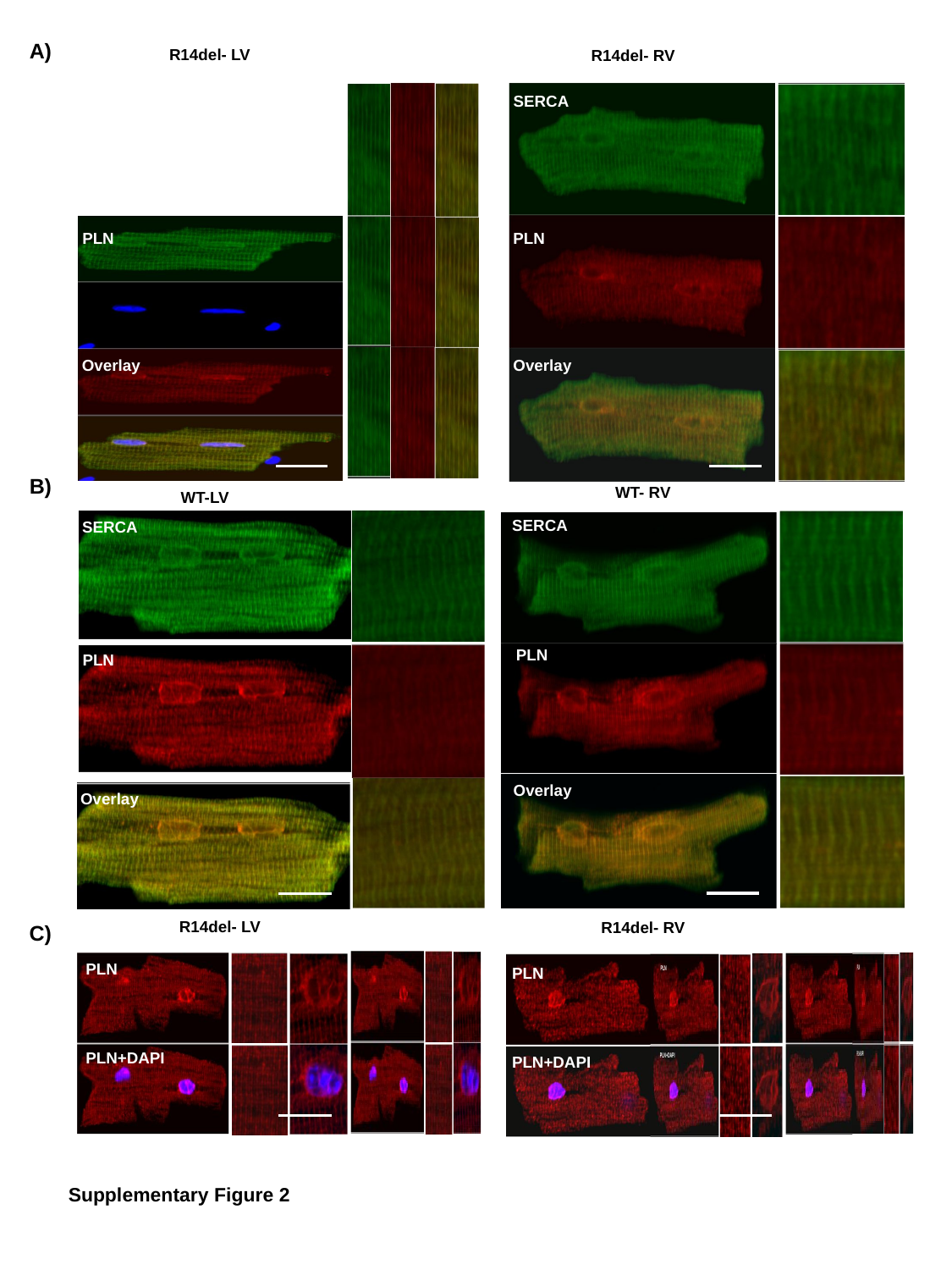

A)
R14del- LV
R14del- RV
WT-LV
SERCA
PLN
Overlay
SERCA
PLN
Overlay
PLN
PLN+DAPI
PLN
PLN+DAPI
SERCA
SERCA
PLN
PLN
Overlay
Overlay
 B)
WT- RV
R14del- LV
R14del- RV
 C)
Supplementary Figure 2

## Slide 4
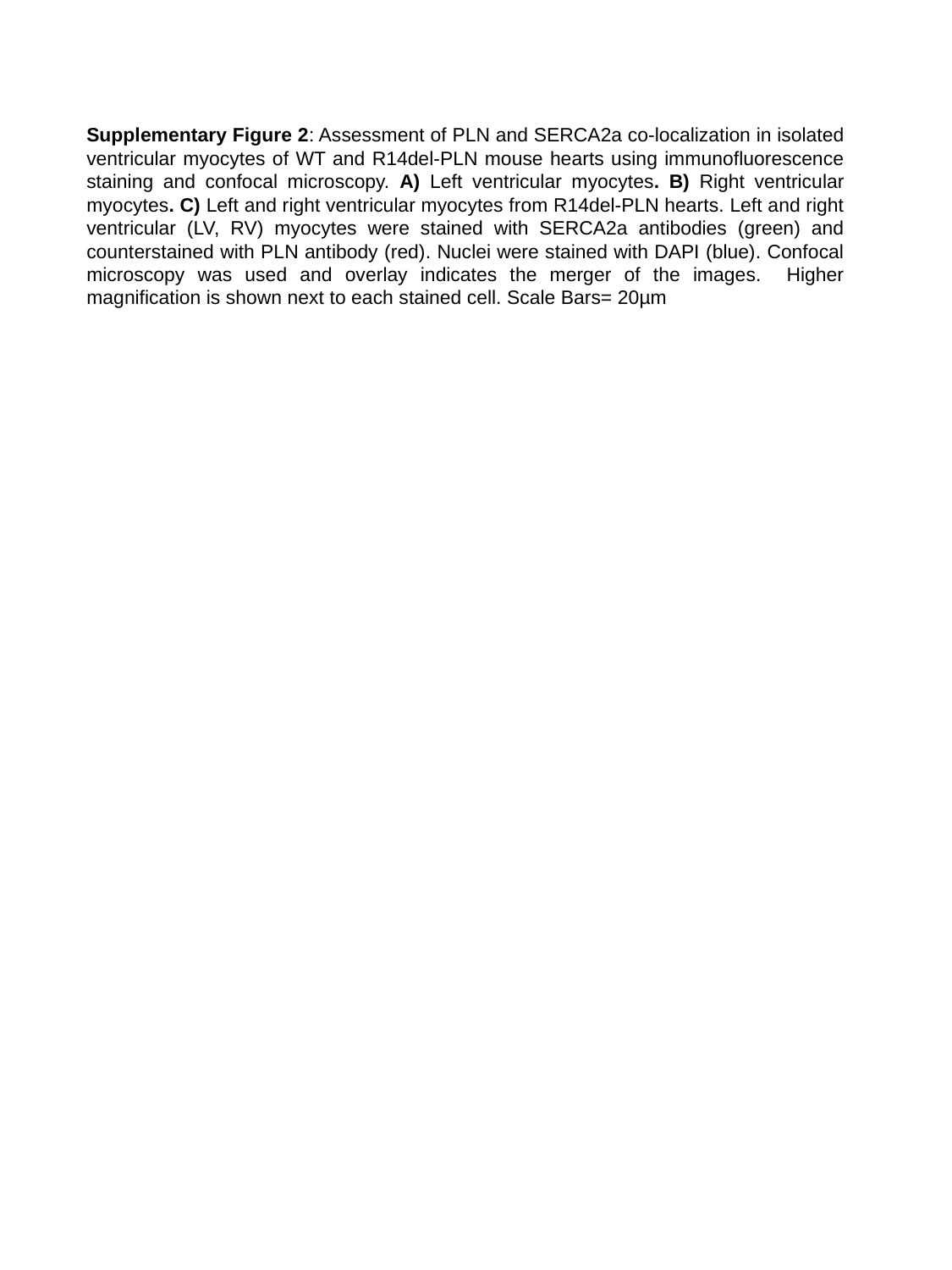

Supplementary Figure 2: Assessment of PLN and SERCA2a co-localization in isolated ventricular myocytes of WT and R14del-PLN mouse hearts using immunofluorescence staining and confocal microscopy. A) Left ventricular myocytes. B) Right ventricular myocytes. C) Left and right ventricular myocytes from R14del-PLN hearts. Left and right ventricular (LV, RV) myocytes were stained with SERCA2a antibodies (green) and counterstained with PLN antibody (red). Nuclei were stained with DAPI (blue). Confocal microscopy was used and overlay indicates the merger of the images. Higher magnification is shown next to each stained cell. Scale Bars= 20µm

## Slide 5
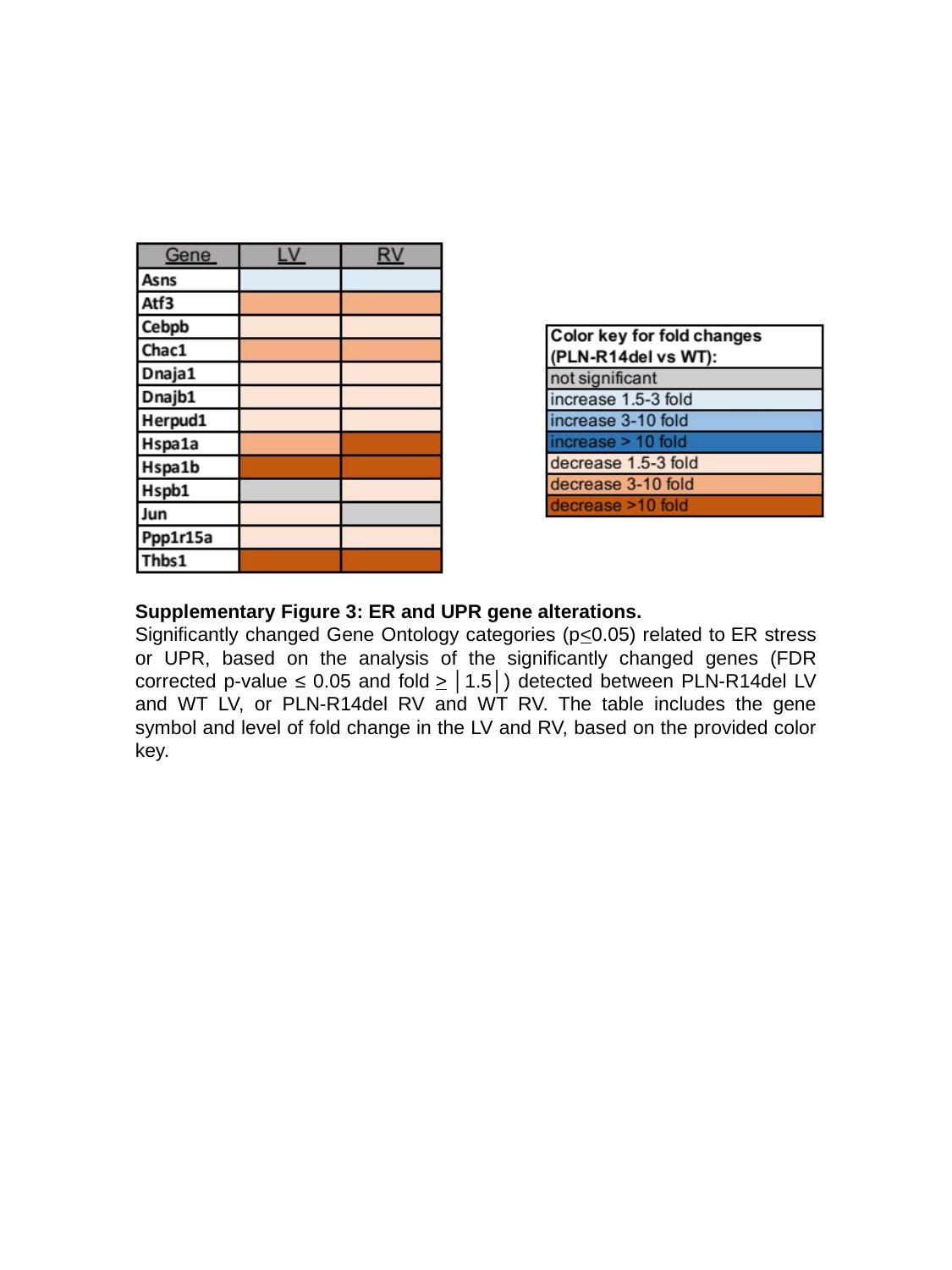

Supplementary Figure 3: ER and UPR gene alterations.
Significantly changed Gene Ontology categories (p<0.05) related to ER stress or UPR, based on the analysis of the significantly changed genes (FDR corrected p-value ≤ 0.05 and fold > │1.5│) detected between PLN-R14del LV and WT LV, or PLN-R14del RV and WT RV. The table includes the gene symbol and level of fold change in the LV and RV, based on the provided color key.

## Slide 6
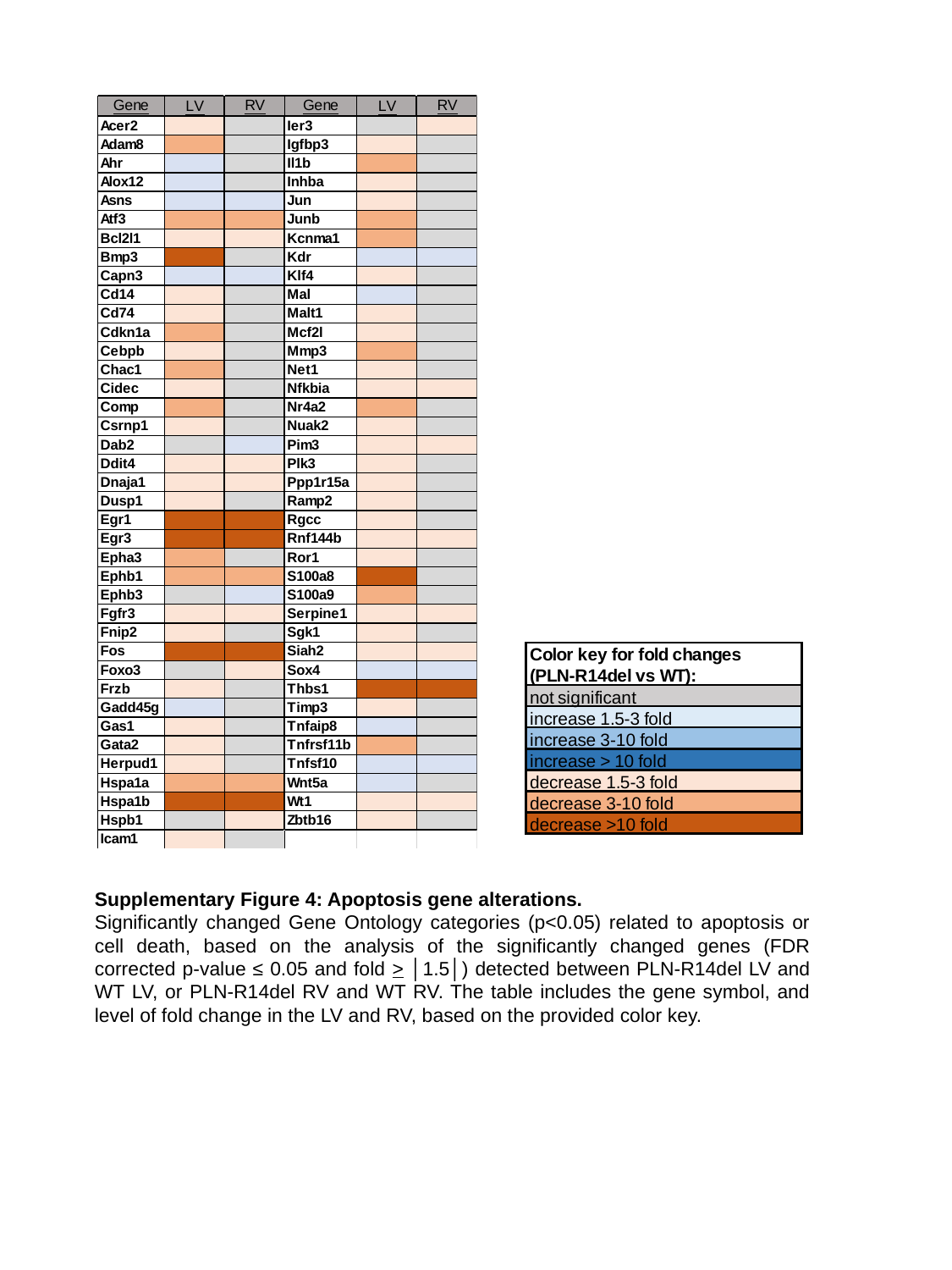

C
o
l
o
r
k
e
y
f
o
r
f
o
l
d
c
h
a
n
g
e
s
(
P
L
N
-
R
1
4
d
e
l
v
s
W
T
)
:
n
o
t
s
i
g
n
i
f
i
c
a
n
t
i
n
c
r
e
a
s
e
1
.
5
-
3
f
o
l
d
i
n
c
r
e
a
s
e
3
-
1
0
f
o
l
d
i
n
c
r
e
a
s
e
>
1
0
f
o
l
d
d
e
c
r
e
a
s
e
1
.
5
-
3
f
o
l
d
d
e
c
r
e
a
s
e
3
-
1
0
f
o
l
d
d
e
c
r
e
a
s
e
>
1
0
f
o
l
d
Supplementary Figure 4: Apoptosis gene alterations.
Significantly changed Gene Ontology categories (p<0.05) related to apoptosis or cell death, based on the analysis of the significantly changed genes (FDR corrected p-value ≤ 0.05 and fold > │1.5│) detected between PLN-R14del LV and WT LV, or PLN-R14del RV and WT RV. The table includes the gene symbol, and level of fold change in the LV and RV, based on the provided color key.
